# Unusually High Levels of n-6 Polyunsaturated Fatty Acids in Whale Sharks and Reef Manta Rays

**DOI:** 10.1007/s11745-013-3829-8

**Published:** 2013-08-22

**Authors:** L. I. E. Couturier, C. A. Rohner, A. J. Richardson, S. J. Pierce, A. D. Marshall, F. R. A. Jaine, K. A. Townsend, M. B. Bennett, S. J. Weeks, P. D. Nichols

**Affiliations:** 1 School of Biomedical Sciences, The University of Queensland, St Lucia, QLD 4072 Australia; 2 Climate Adaptation Flagship, CSIRO Marine and Atmospheric Research, Dutton Park, QLD 4102 Australia; 3 Manta Ray and Whale Shark Research Centre, Marine Megafauna Foundation, Praia do Tofo, Inhambane, Mozambique; 4 Biophysical Oceanography Group, School of Geography, Planning and Environmental Management, The University of Queensland, St Lucia, QLD 4072 Australia; 5 Centre for Applications in Natural Resource Mathematics, The University of Queensland, St Lucia, QLD 4072 Australia; 6 Wild Me, Praia do Tofo, Inhambane, Mozambique; 7 School of Biological Sciences, The University of Queensland, St Lucia, QLD 4072 Australia; 8 Wealth from Oceans Flagship, CSIRO Marine and Atmospheric Research, Hobart, TAS 7000 Australia

**Keywords:** n-3 Fatty acids, Arachidonic acid, Planktivores, Zooplankton, Elasmobranch

## Abstract

Fatty acid (FA) signature analysis has been increasingly used to assess dietary preferences and trophodynamics in marine animals. We investigated FA signatures of connective tissue of the whale shark *Rhincodon typus* and muscle tissue of the reef manta ray *Manta alfredi*. We found high levels of n-6 polyunsaturated fatty acids (PUFA), dominated by arachidonic acid (20:4n-6; 12–17 % of total FA), and comparatively lower levels of the essential n-3 PUFA—eicosapentaenoic acid (20:5n-3; ~1 %) and docosahexaenoic acid (22:6n-3; 3–10 %). Whale sharks and reef manta rays are regularly observed feeding on surface aggregations of coastal crustacean zooplankton during the day, which generally have FA profiles dominated by n-3 PUFA. The high levels of n-6 PUFA in both giant elasmobranchs raise new questions about the origin of their main food source.

## Introduction

The whale shark *Rhincodon typus* and the reef manta ray *Manta alfredi* are giant planktivorous elasmobranchs that are presumed to feed predominantly on aggregations of zooplankton in highly productive areas [1, 2]. Direct studies on the diet of these elasmobranchs are limited to examination of a few stomach contents, faecal material and stable isotope analyses [3–6], while recent field observations suggest that their diets are mostly composed of crustacean zooplankton [1, 7]. It is unknown, however, whether near-surface zooplankton are a major or only a minor part of their diets, whether these large elasmobranchs target other prey, or whether they feed in areas other than surface waters along productive coastlines.

Here we used signature fatty acid (FA) analysis to assess dietary preferences of *R. typus* and *M. alfredi*. The essential long-chain (≥C_20_) polyunsaturated fatty acids (LC-PUFA) of fishes are most likely derived directly from the diet, as higher consumers generally lack the ability to biosynthesise these FA de novo [8, 9]. The fatty acid profile of zooplankton is usually dominated by PUFA with a high n-3/n-6 ratio, and generally contains high levels of eicosapentaenoic acid (EPA, 20:5n-3) and/or docosahexaenoic acid (DHA, 22:6n-3) [8, 10, 11]. Considering this, it was expected that FA profiles of *R. typus* and *M. alfredi* tissues would be similarly n-3 PUFA dominated.

## Materials and Methods

Tissue samples were collected from live, unrestrained specimens in southern Mozambique (14 *R. typus* and 12 *M. alfredi*) and eastern Australia (9 *M. alfredi*) using a modified Hawaiian hand-sling with a fitted biopsy needle tip between June–August 2011. Biopsies of *R. typus* were extracted laterally between the 1st and 2nd dorsal fin and penetrated ~20 mm deep from the skin into the underlying connective tissue. Biopsies of *M. alfredi* were of similar size, but were mainly muscle tissue, extracted from the ventro-posterior area of the pectoral fins away from the body cavity. Biopsies were immediately put on ice in the field and then stored at −20 °C for up to 3 months before analysis.

Lipids were extracted overnight using the modified Bligh and Dyer [12] method with a one-phase methanol:chloroform:water (2:1:0.8 by volume) mixture. Phases were separated by adding water and chloroform, followed by rotary evaporation of the chloroform in vacuo at ~40 °C. Total lipid extracts were concentrated by application of a stream of inert nitrogen gas and samples were stored in chloroform at −20 °C before FA analysis.

The total lipid extract from each sample was spotted on chromarods that were developed for 25 min in a polar solvent system (hexane:diethyl-ether:acetic acid, 60:17:0.1 by volume). The chromarods were then dried in an oven for 10 min at 100 °C and analysed immediately. Lipid class composition was determined for each sample using an Iatroscan Mark V TH10 thin layer chromatograph combined with a flame ionisation detector. A standard solution containing wax esters, triacylglycerol, free FA, sterols and phospholipids (Nu-Chek Prep. Inc., MN, USA) was run with the samples. Each peak was identified by comparison of Rf with the standard chromatogram. Peak areas were measured using SIC-480II Iatroscan™ Integrating Software v.7.0-E (System Instruments Co., Mitsubishi Chemical Medicine Corp., Japan) and quantified to mass per μL spotted using predetermined linear regressions.

An aliquot of the total extracted lipids was treated with methanol:hydrochloric acid:chloroform (10:1:1), heated at ~80 °C for 2 h and the resulting fatty acid methyl esters were extracted into hexane:chloroform (4:1). Samples were analysed using an Agilent Technologies 7890 B gas chromatography (GC) (Palo Alto, California, USA) equipped with a non-polar Equity™-1 fused silica capillary column (15 m × 0.1 mm i.d., 0.1 μm film thickness), a flame ionisation detector, a split/split-less injector and an Agilent Technologies 7683 B Series auto sampler. Helium was the carrier gas. Samples were injected in split-less mode at an oven temperature of 120 °C. After injection, oven temperature was raised to 270 °C at 10 °C/min and finally to 300 °C at 5 °C/min. Peaks were quantified with Agilent Technologies ChemStation software (Palo Alto, California, USA). Sterols were also separated under the GC conditions used, and largely comprised cholesterol. GC results typically have an error of up to ±5 % of individual component area. Peak identities were confirmed with a Finnigan ThermoQuest GCQ GC mass-spectrometer (GC-MS) system (Finnigan, San Jose,CA) [13]. Percentage FA data were calculated from the areas of chromatogram peaks. All FA are expressed as mole percentage of total FA.

## Results and Discussion

Fatty acids of both *M. alfredi* muscle tissue and *R. typus* connective tissue were predominantly derived from phospholipids (Table 1). The classes of phospholipids were not distinguished in this study, but should be examined in future studies where phospholipids are found to be the dominant lipid class of these two giant elasmobranchs. The FA profile of *M. alfredi* was dominated by PUFA (34.9 % of total FA), while saturated FA were most abundant in *R. typus* (39.1 % of total FA) (Table 2). The main FA in both species included 18:0, 18:1n-9, 16:0 and 20:4n-6. Arachidonic acid (AA; 20:4n-6) was the most abundant FA in *R. typus* (16.9 %) whereas 18:0 was most abundant in *M. alfredi* (16.8 %). Both species had a relatively low level of EPA (1.1 and 1.2 %) and *M. alfredi* had a relatively high level of DHA (10.0 %) compared to *R. typus* (2.5 %). Fatty acid signatures of *R. typus* and *M. alfredi* were different to expected profiles of species that feed predominantly on crustacean zooplankton, which are typically dominated by n-3 PUFA and have high levels of EPA and/or DHA [8, 10, 11]. Instead, profiles of both large elasmobranchs were dominated by n-6 PUFA (>20 % total FA), with an n-3/n-6 ratio <1 and markedly high levels of AA (Table 2). The FA profiles of *M. alfredi* were broadly similar between the two locations, although some differences were observed that are likely due to dietary differences. Future research should aim to look more closely at these differences and potential dietary contributions.

**Table 1:** Means ± SE (standard error) lipid class compositions of whale shark (*n* = 14) and reef manta ray (*n* = 15) tissue samples, expressed as % of total lipid

Lipid class	Whale shark (*n* = 14)	Reef manta ray (*n* = 15)
	% Total lipid ± SE	% Total lipid ± SE
WE	2.8 ± 1.3	0.6 ± 0.4
TAG	3.3 ± 1.4	3.4 ± 0.7
FFA	5.3 ± 1.0	2.1 ± 0.3
ST	20.5 ± 0.8	10.8 ± 1.1
PL	68.1 ± 3.5	83.0 ± 1.5
Total lipid content (mg g^−1^)	1.8 ± 1.1	3.8 ± 0.3

Total lipid content is expressed as mg g^−1^ of tissue wet mass

*WE* wax esters, *TAG* triacylglycerols, *FFA* free fatty acids, *ST* sterols (comprising mostly cholesterol), *PL* phospholipids

**Table 2:** FA composition (mol% of total FA) of the whale shark *R. typus* (*n* = 14) and the reef manta ray *M. alfredi* (*n* = 21) [minor fatty acids (≤1 %) are not shown]

	*R. typus*	*M. alfredi*
	Mean (±SEM)	Mean (±SEM)
∑SFA	39.1 (0.7)	35.1 (0.7)
16:0	13.8 (0.5)	14.7 (0.4)
17:0	1.6 (0.1)	0
i18:0	1.1 (0.1)	0.3 (0.1)
18:0	17.8 (0.5)	16.8 (0.4)
∑MUFA	31.0 (0.9)	29.9 (0.7)
16:1n-7c	2.1 (0.3)	2.7 (0.3)
17:1n-8c^a^	1.8 (0.3)	0.7 (0.1)
18:1n-9c	16.7 (0.7)	15.7 (0.4)
18:1n-7c	4.6 (0.5)	6.1 (0.2)
20:1n-9c	0.7 (0.02)	1.0 (0.03)
24:1n-9c	1.9 (0.1)	1.1 (0.1)
∑PUFA	29.9 (0.9)	34.9 (1.2)
∑n-3	6.1 (0.3)	13.4 (0.6)
20:5n-3 (EPA)	1.1 (0.1)	1.2 (0.1)
22:6n-3 (DHA)	2.5 (0.2)	10.0 (0.5)
22:5n-3	2.1 (0.1)	2.0 (0.1)
∑n-6	23.8 (0.8)	21.0 (1.4)
20:4n-6 (AA)	16.9 (0.6)	11.7 (0.8)
22:5n-6	0.9 (0.1)	3.3 (0.3)
22:4n-6	5.5 (0.3)	5.1 (0.5)
n-3/n-6	0.3 (0.02)	0.7 (0.1)

*SFA* saturated fatty acids, *MUFA* monounsaturated fatty acids, *PUFA* polyunsaturated fatty acids, *EPA* eicosapentaenoic acid, *DHA* docosahexaenoic acid, *AA* arachidonic acid

^a^Includes a17:0 coeluting

The n-6-dominated FA profiles are rare among marine fishes. Most other large pelagic animals and other marine planktivores have an n-3-dominated FA profile and no other chondrichthyes investigated to date has an n-3/n-6 ratio <1 [14–16] (Table 3, literature data are expressed as wt%). The only other pelagic planktivore with a similar n-3/n-6 ratio (i.e. 0.9) is the leatherback turtle, that feeds on gelatinous zooplankton [17]. Only a few other marine species, such as several species of dolphins [18], benthic echinoderms and the bottom-dwelling rabbitfish *Siganus nebulosus* [19], have relatively high levels of AA, similar to those found in whale sharks and reef manta rays (Table 3).

**Table 3:** Polyunsaturated fatty acid composition of chondrichthyan, planktivore, large pelagic and detrivore species

Species	Feeding habitat	Tissue	∑n-3	∑n-6	AA	EPA	DHA	n-3/n-6	Reference
Whale shark—*R. typus* (mol%)	Epipelagic—planktivore	Skin	6.1	23.8	16.9	1.1	2.5	0.3	This study
Whale shark—*R. typus* (wt%)	Epipelagic—planktivore	Skin	6.7	25.4	17.8	1.2	2.8	0.3	This study
Reef manta ray—*M. alfredi* (mol%)	Epipelagic—planktivore	Muscle	13.4	21.0	11.7	1.2	10.0	0.7	This study
Reef manta ray—*M. alfredi* (wt%)	Epipelagic—planktivore	Muscle	14.9	21.6	11.8	1.2	11.3	0.7	This study
Other chondrichthyes									
Port Jackson shark—*Heterodontus portusjacksoni*	Demersal—carnivore	Muscle	23.6	19.4	13.8	3.7	15.4	1.2	[45]
Sandy-backed stingaree—*Urolophus bucculentus*	Demersal—carnivore	Muscle	32.9	16.5	12.6	3.1	27.9	2.0	[45]
Southern chimaera—*Chimaera fulva*	Deep sea—carnivore	Muscle	30.4	11.2	4.7	3.4	23.3	2.7	[46]
Angel shark—*Squatina australis*	Demersal—carnivore	Muscle	45.2	10.5	7.6	6.1	36.5	4.3	[45]
Longnose velvet dogfish—*Centroselachus crepidater*	Deep sea—carnivore	Muscle	39.1	6.6	4.4	2.3	32.2	5.9	[46]
Shortnose spurdog—*Squalus megalops*	Deep sea—carnivore	Muscle	37.5	6.4	3.6	1.2	32.3	5.9	[46]
South China catshark—*Apristurus sinensis*	Deep sea—carnivore	Muscle	38.5	6.4	3.4	2.9	28.9	6	[46]
Broadnose sevengill shark—*Notorynchus cepedianus*	Deep sea—carnivore	Liver	23.2	3.2	1.7	3.4	16.6	7.2	[46]
Planktivores									
Leatherback turtle—*Dermochelys coriacea*	Epipelagic—planktivore	Muscle	15.5	17.3	15.5	6.1	5.7	0.9	[17]
Jellyfish—*Aurelia* sp.	Epipelagic—planktivore	Whole	34.5	12.2	9.9	14.1	9.8	2.8	[25]
Finwhale—*Balaenoptera physalus*	Pelagic—planktivore	Blubber oil	6.7	2.3	0.3	1.8	2.74	2.9	[47]
Anchovies—*Engraulis mordax mordax*	Pelagic—planktivore	Whole	22.9	4.9	0.4	13.5	8.8	27.8	[48]
Large pelagics									
Dolphin—*mixed species*	Epipelagic—carnivore	Muscle	16.3	18.6	14.2	6.4	7.6	0.9	[18]
Gray whale—*E. robustus*	Pelagic—planktivore	Muscle			4.7	7.5	1.2	~1.8	[49]
Ocean sunfish—*Mola mola*	Pelagic—carnivore	Muscle	29.4	10.8	7.73	8.8	17.0	2.7	[50]
Benthic feeders									
Sea cucumber—*Holothuria scabra*	Benthic—deposit feeder	Whole	10.7	22.6	19.1	8.2	1.5	0.5	[19]
Sea urchin—*Heliocidaris erythrogramma*	Benthic—deposit feeder	Whole	10.7	14.6	6.1	8.3	0.4	0.7	[19]
Dusky rabbitfish—*Siganus nebulosus*	Benthic—deposit feeder	Muscle	18.5	20.5	12.4	1.3	14.6	0.9	[19]

Data from this study for *Rhincodon typus* and *Manta alfredi* are expressed in both mol% and wt% format, with all literature data as wt%

*EPA* eicosapentaenoic acid, *DHA* docosahexaenoic acid, *AA* arachidonic acid

The trophic pathway for n-6-dominated FA profiles in the marine environment is not fully understood. Although most animal species can, to some extent, convert linoleic acid (LA, 18:2n-6) to AA [8], only traces of LA (<1 %) were present in the two filter-feeders here. Only marine plant species are capable of biosynthesising long-chain n-3 and n-6 PUFA de novo, as most animals do not possess the enzymes necessary to produce these LC-PUFA [8, 9]. These findings suggest that the origin of AA in *R. typus* and *M. alfredi* is most likely directly related to their diet.

Although FA are selectively incorporated into different elasmobranch tissues, little is known on which tissue would best reflect the diet FA profile. McMeans et al. [14] recently showed that FA profile of muscle in the Greenland shark is the most representative of its prey FA profiles. It is thus assumed here that the muscle tissue of *M. alfredi* is representative of its diet, but the extent to which the FA profile of the subdermal connective tissue of *R. typus* reflects its diet is unknown.

Certain species of phytoplankton including diatoms, and some macro algae such as Rhodophyta can biosynthesise n-6 PUFA, with levels of over 40 % (as wt%) of AA recorded [20, 21]. Although phytoplankton and macro algae have been reported in *R. typus* stomach contents, they are assumed to be incidentally ingested [22]. The feeding apparatus and feeding strategy of *R. typus* and *M. alfredi* are adapted for targeting larger prey [23, 24]. There is no observational evidence of either species targeting phytoplankton, but there are frequent observations of feeding on zooplankton patches. More plausibly, n-6 LC-PUFA from phytoplankton could enter the food chain when consumed by zooplankton and subsequently be transferred to higher-level consumers. It is unclear what type of zooplankton is likely to feed on AA-rich algae. To date, only a few jellyfish species are known to contain high levels of AA (2.8–9.9 % of total FA as wt%), but they also have high levels of EPA, which are low in *R. typus* and *M. alfredi* [17, 25, 26].

Some protozoans and microeukaryotes, including heterotrophic thraustochytrids in marine sediments are rich in AA [27–30] and could be linked with high n-6 LC-PUFA and AA levels in benthic feeders (n-3/n-6 = 0.5–0.9; AA = 6.1–19.1 % as wt%; Table 3), such as echinoderms, stingrays and other benthic fishes. However, the pathway of utilisation of AA from these micro-organisms remains unresolved. *R. typus* and *M. alfredi* may feed close to the sea floor and could ingest sediment with associated protozoan and microeukaryotes suspended in the water column; however, they are unlikely to target such small sediment-associated benthos. The link to *R. typus* and *M. alfredi* could be through benthic zooplankton, which potentially feed within the sediment on these AA-rich organisms and then emerge in high numbers out of the sediment during their diel vertical migration [31, 32]. It is unknown to what extent *R. typus* and *M. alfredi* feed at night when zooplankton in shallow coastal habitats emerges from the sediment.

The subtropical/tropical distribution of *R. typus* and *M. alfredi* is likely to partly contribute to their n-6-rich PUFA profiles. Although still strongly n-3-dominated, the n-3/n-6 ratio in fish tissue noticeably decreases from high to low latitudes, largely due to an increase in n-6 PUFA, particularly AA (Table 3) [33–35]. This latitudinal effect alone does not, however, explain the unusual FA signatures of *R. typus* and *M. alfredi*.

We found that *M. alfredi* contained more DHA than EPA, while *R. typus* had low levels of both these n-3 LC-PUFA, and there was less of either n-3 LC-PUFA than AA in both species. As DHA is considered a photosynthetic biomarker of a flagellate-based food chain [8, 10], high levels of DHA in *M. alfredi* could be attributed to crustacean zooplankton in the diet, as some zooplankton species feed largely on flagellates [36]. By contrast, *R. typus* had low levels of EPA and DHA, and the FA profile showed AA as the major component.

Our results suggest that the main food source of *R. typus* and *M. alfredi* is dominated by n-6 LC-PUFA that may have several origins. Large, pelagic filter-feeders in tropical and subtropical seas, where plankton is scarce and patchily distributed [37], are likely to have a variable diet. At least for the better-studied *R. typus*, observational evidence supports this hypothesis [38–43]. While their prey varies among different aggregation sites [44], the FA profiles shown here suggest that their feeding ecology is more complex than simply targeting a variety of prey when feeding at the surface in coastal waters. Trophic interactions and food web pathways for these large filter-feeders and their potential prey remain intriguingly unresolved. Further studies are needed to clarify the disparity between observed coastal feeding events and the unusual FA signatures reported here, and to identify and compare FA signatures of a range of potential prey, including demersal and deep-water zooplankton.

## Abbreviations

ARA: Arachidonic acid
DHA: Docosahexaenoic acid
EPA: Eicosapentaenoic acid
FA: Fatty acid(s)
GC: Gas chromatography
LA: Linoleic acid
LC-PUFA: Long chain- polyunsaturated fatty acid(s)
MUFA: Monounsaturated fatty acid(s)
PUFA: Polyunsaturated fatty acid(s)
SEM: Standard error of the mean
SFA: Saturated fatty acid(s)

## References

[1] Nelson JD, Eckert SA (2007). Foraging ecology of whale sharks (*Rhincodon typus*) within Bahía De Los Angeles, Baja California Norte, México. Fish Res.

[2] Anderson RC, Adam MS, Goes JI (2011). From monsoons to mantas: seasonal distribution of *Manta alfredi* in the Maldives. Fish Oceanogr.

[3] Jarman S, Wilson S (2004). DNA-based species identification of krill consumed by whale sharks. J Fish Biol.

[4] Silas E, Rajagopalan M (1963). On a recent capture of a whale shark (*Rhincodon typus* Smith) at Tuticorin, with a note on information to be obtained on whale sharks from Indian waters. J Mar Biol Ass India.

[5] Borrell A, Cardona L, Kumarran RP, Aguilar A (2011). Trophic ecology of elasmobranchs caught off Gujarat, India, as inferred from stable isotopes. ICES J Mar Sci.

[6] Whitley GP (1936). The Australian devil ray, *Daemomanta alfredi* (Krefft), with remarks on the superfamily Mobuloidae (order Batoidei). Aust Zool.

[7] Papastamatiou YP, DeSalles PA, McCauley DJ (2012). Area-restricted searching by manta rays and their response to spatial scale in lagoon habitats. Mar Ecol Prog Ser.

[8] Dalsgaard J, St John M, Kattner G, Müller-Navarra D, Hagen W (2003). Fatty acid trophic markers in the pelagic marine environment. Adv Mar Biol.

[9] Iverson SJ, Arts MT, Brett MT, Kainz M (2009). Tracing aquatic food webs using fatty acids: from qualitative indicators to quantitative determination. Lipids in aquatic ecosystems.

[10] Brett MT, Müller-Navarra DC, Persson J, Arts MT, Bakes MJ, Kainz M (2009). Crustacean zooplankton fatty acid composition. Lipids in aquatic ecosystems.

[11] Sargent J, Falk-Petersen S (1988). The lipid biochemistry of calanoid copepods. Hydrobiologia.

[12] Bligh EG, Dyer WJ (1959). A rapid method of total lipid extraction and purification. Can J Biochem Physiol.

[13] Phleger CF, Nelson MM, Mooney BD, Nichols PD, Ritar AJ, Smith GG, Hart PR, Jeffs AG (2001). Lipids and nutrition of the southern rock lobster, *Jasus edwardsii*, from hatch to Puerulus. Mar Freshw Res.

[14] McMeans BC, Arts MT, Fisk AT (2012). Similarity between predator and prey fatty acid profiles is tissue dependent in Greenland sharks (*Somniosus microcephalus*): implications for diet reconstruction. J Exp Mar Biol Ecol.

[15] Pethybridge H, Daley RK, Nichols PD (2011). Diet of demersal sharks and chimaeras inferred by fatty acid profiles and stomach content analysis. J Exp Mar Biol Ecol.

[16] Schaufler L, Heintz R, Sigler M, Hulbert L (2005). Fatty acid composition of sleeper shark (*Somniosus pacificus*) liver and muscle reveals nutritional dependence on Planktivores.

[17] Holland D, Davenport J, East J (1990). The fatty acid composition of the leatherback turtle *Dermochelys coriacea* and its jellyfish prey. J Mar Biol Assoc UK.

[18] Williams G, Crawford M, Perrin W (1987). Comparison of the fatty acid component in structural lipids from dolphins, zebra and giraffe: possible evolutionary implications. J Zool.

[19] Nichols PD, Mooney BD, Elliott NG (2002). Nutritional value of Australian seafood ii.

[20] Dunstan GA, Volkman JK, Barrett SM, Leroi JM, Jeffrey S (1994). Essential polyunsaturated fatty acids from 14 species of diatom (Bacillariophyceae). Phytochem.

[21] Johns R, Nichols P, Perry G (1979). Fatty acid composition of ten marine algae from Australian waters. Phytochemistry.

[22] Colman JG (1997). A review of the biology and ecology of the whale shark. J Fish Biol.

[23] Motta PJ, Maslanka M, Hueter RE, Davis RL, De La Parra R, Mulvany SL, Habegger ML, Strother JA, Mara KR, Gardiner JM (2010). Feeding anatomy, filter-feeding rate, and diet of whale sharks *Rhincodon typus* during surface ram filter feeding off the Yucatan Peninsula, Mexico. Zoology.

[24] Paig-Tran EWM, Bizzarro JJ, Strother JA, Summers AP (2011). Bottles as models: predicting the effects of varying swimming speed and morphology on size selectivity and filtering efficiency in fishes. J Exp Biol.

[25] Nichols PD, Danaher KT, Koslow JA (2003). Occurrence of high levels of tetracosahexaenoic acid in the jellyfish *Aurelia* sp.. Lipids.

[26] Sipos J, Ackman RG (1968). Jellyfish (*Cyanea capillata*) lipids: fatty acid composition. J Fish Res Board Can.

[27] Bell M, Sargent J (1985). Fatty acid analyses of phosphoglycerides from tissues of the clam *Chlamys Islandica* (Muller) and the starfish *Ctenodiscus crispatus* (Retzius) from Balsfjorden, Northern Norway. J Exp Mar Biol Ecol.

[28] Howell KL, Pond DW, Billett DSM, Tyler PA (2003). Feeding ecology of deep-sea seastars (Echinodermata: Asteroidea): a fatty-acid biomarker approach. Mar Ecol Prog Ser.

[29] Nichols DS (2003). Prokaryotes and the input of polyunsaturated fatty acids to the marine food web. FEMS Microbiol Lett.

[30] Lee Chang KJ, Dunstan GA, Abell GCJ, Clementson LA, Blackburn SI, Nichols PD, Koutoulis A (2012). Biodiscovery of new Australian thraustochytrids for production of biodiesel and long-chain omega-3 oils. Appl Microbiol Biotechnol.

[31] Hutchinson GE (1967). A treatise on limnology.

[32] Stoecker DK, Capuzzo JMD (1990). Predation on protozoa: its importance to zooplankton. J Plankton Res.

[33] Armstrong SG, Wyllie SG, Leach DN (1994). Effects of season and location of catch on the fatty acid compositions of some Australian fish species. Food Chem.

[34] Sinclair A, O’Dea K, Naughton J, Sutherland T, Wankowski J (1984) Polyunsaturated fatty acid types in some Australian and Antarctic fish. In: Proceedings of the nutrition society of Australia conference 9:188

[35] Gibson R (1983). Australian fish—an excellent source of both arachidonic acid and n-3 polyunsaturated fatty acids. Lipids.

[36] Kleppel GS (1993). On the diets of calanoid copepods. Mar Ecol Prog Ser.

[37] Lalli CM, Parsons TR (1997). Biological oceanography: an introduction.

[38] de la Parra Venegas R, Hueter R, Cano JG, Tyminski J, Remolina JG, Maslanka M, Ormos A, Weigt L, Carlson B, Dove A (2011). An unprecedented aggregation of whale sharks, *Rhincodon typus*, in Mexican coastal waters of the Caribbean sea. PLoS One.

[39] Duffy C (2002). Distribution, seasonality, lengths, and feeding behaviour of whale sharks (*Rhincodon typus*) observed in New Zealand waters. N Z J Mar Freshw Res.

[40] Heyman WD, Graham RT, Kjerfve B, Johannes RE (2001). Whale sharks *Rhincodon typus* aggregate to feed on fish spawn in Belize. Mar Ecol Prog Ser.

[41] Robinson DP, Jaidah MY, Jabado RW, Lee-Brooks K, El-Din NMN, Malki AAA, Elmeer K, McCormick PA, Henderson AC, Pierce SJ (2013). Whale sharks, *Rhincodon typus*, aggregate around offshore platforms in Qatari waters of the Arabian Gulf to feed on fish spawn. PLoS One.

[42] Rowat D, Meekan M, Engelhardt U, Pardigon B, Vely M (2007). Aggregations of juvenile whale sharks (*Rhincodon typus*) in the Gulf of Tadjoura, Djibouti. Environ Biol Fishes.

[43] Meekan M, Jarman S, McLean C, Schultz M (2009). DNA evidence of whale sharks (*Rhincodon typus*) feeding on red crab (*Gecarcoidea natalis*) larvae at Christmas Island, Australia. Mar Freshw Res.

[44] Rowat D, Brooks K (2012). A review of the biology, fisheries and conservation of the whale shark *Rhincodon typus*. J Fish Biol.

[45] Dunstan GA, Sinclair AJ, O’Dea K, Naughton JM (1988). The lipid content and fatty acid composition of various marine species from southern Australian coastal waters. Comp Biochem Physiol B.

[46] Pethybridge H, Daley R, Virtue P, Nichols P (2010). Lipid composition and partitioning of deepwater chondrichthyans: inferences of feeding ecology and distribution. Mar Biol.

[47] Ackman RG, Epstein S, Eaton CA (1971). Differences in the fatty acid compositions of blubber fats from Northwestern Atlantic finwhales (*Balaenoptera physalus*) and harp seals (*Pagophilus groenlandica*). Comp Biochem Physiol B.

[48] Lewis R (1967). Fatty acid composition of some marine animals from various depths. J Fish Res Board Can.

[49] Caraveo-Patiño J, Wang Y, Soto LA, Ghebremeskel K, Lehane C, Crawford MA (2009). Eco-physiological repercussions of dietary arachidonic acid in cell membranes of active tissues of the gray whale. Mar Ecol.

[50] Hooper S, Paradis M, Ackman RG (1973). Distribution of *trans*-6-hexadecenoic acid, 7-methyl-7-hexadecenoic acid and common fatty acids in lipids of the ocean sunfish *Mola mola*. Lipids.

